# Impact of hip fracture on all‐cause mortality in Japanese patients with type 2 diabetes mellitus: The Fukuoka Diabetes Registry

**DOI:** 10.1111/jdi.13076

**Published:** 2019-06-12

**Authors:** Yuji Komorita, Masanori Iwase, Yasuhiro Idewaki, Hiroki Fujii, Toshiaki Ohkuma, Hitoshi Ide, Tamaki Jodai‐Kitamura, Masahito Yoshinari, Ai Murao‐Kimura, Yutaro Oku, Udai Nakamura, Takanari Kitazono

**Affiliations:** ^1^ Department of Medicine and Clinical Science Graduate School of Medical Sciences Kyushu University Fukuoka Japan; ^2^ Division of Internal Medicine Fukuoka Dental College Fukuoka Japan; ^3^ Diabetes Center Hakujyuji Hospital Fukuoka Japan; ^4^ Center for Cohort Studies Graduate School of Medical Sciences Kyushu University Fukuoka Japan; ^5^ The George Institute for Global Health University of New South Wales Sydney New South Wales Australia; ^6^ Division of General Internal Medicine School of Oral Health Science Kyushu Dental University Kitakyushu Japan

**Keywords:** Death, Hip fracture, Type 2 diabetes

## Abstract

**Aims/Introduction:**

Patients with type 2 diabetes mellitus have an increased hip fracture risk. We investigated the relationship between hip fracture and all‐cause death in patients with type 2 diabetes in comparison with cardiovascular disease (CVD) or end‐stage renal disease (ERSD).

**Materials and Methods:**

In total, 4,923 Japanese participants with type 2 diabetes (mean age 65 years, 2,790 men, 2,133 women) were followed for a median of 5.3 years (follow‐up rate 99.5%). We evaluated the associations between the presence of hip fracture (*n* = 110), upper limb fracture (*n* = 801), CVD (*n* = 1,344), ESRD (*n* = 104) and all‐cause death by logistic regression analysis.

**Results:**

A total of 309 participants died during follow up. Multivariate‐adjusted odds ratios (ORs) for all‐cause mortality were significantly higher in participants with hip fractures than those without hip fractures (OR 2.67, 95% confidence interval [CI] 1.54–4.41), whereas the ORs for upper limb fracture were not significant. The ORs for all‐cause mortality were significantly higher in participants with CVD than those without CVD (OR 1.78, 95% CI, 1.39–2.70) and ESRD (OR 2.36, 95% CI 1.32–4.05). The ORs for all‐cause mortality of hip fracture were not affected by further adjustment for CVD and ESRD (OR 2.74, 95% CI 1.58–4.54). The cause of death was infection (40.0%), malignant neoplasm (25.0%) and CVD (15.0%) among participants with hip fracture.

**Conclusions:**

Hip fractures were associated with an increased risk of death among Japanese patients with type 2 diabetes, independently of CVD and ESRD.

## Graphical abstract

Hip fracture is associated with increased mortality in the general population, although few studies have investigated the impact of hip fractures on the risk of death in patients with type 2 diabetes who also have a higher prevalence of fatal diseases, such as cardiovascular disease, renal disease or malignant neoplasia. In this study, the presence of hip fracture was associated with an increased risk of death among Japanese patients with type 2 diabetes, independently of cardiovascular disease and end‐stage renal disease. It should be emphasized that hip fracture is a critical event in the aging population of patients with type 2 diabetes.

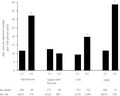

## Introduction

The recent epidemic of diabetes mellitus, along with advancements in the treatment of diabetes and its complications, has led to a rapid increase in the number of aged patients with diabetes^1^, ^2^, ^3^. A better understanding of geriatrics is required in the clinical management of patients with diabetes^4^. Osteoporosis occurs with aging and increases the risk of fragility fractures, which cause other comorbidities or increased mortality in persons of advanced age^5^. Epidemiological studies have shown an increased risk of hip or other fractures in patients with type 2 diabetes than those without type 2 diabetes^6^. This is partly explained by non‐enzymatic glycation of the collagen within bones, decreased bone turnover, oxidative stress and adverse effects of certain diabetes medications^6^, ^7^.

Fragility fractures, especially hip fractures, are associated with increased mortality^5^, ^8^, ^9^, ^10^, ^11^, ^12^. Many factors are associated with the higher mortality rates after hip fracture, including older age, poor physical and cognitive function, comorbid conditions, frailty, and postoperative complications, such as cardiac and pulmonary complications, infections, and an increased risk of thromboembolism, most of which are common in patients with type 2 diabetes. However, few studies have investigated the impact of hip fractures on the risk of death in patients with type 2 diabetes who also have a higher prevalence of fatal diseases, such as cardiovascular disease (CVD)^13^, renal disease^14^ or malignant neoplasia^15^. Because the cause of death in patients with diabetes varies by country and ethnicity, it is important to study the impact of hip fractures on mortality in each country or ethnicity. In this context, we investigated the relationship between hip fracture and all‐cause death in a hospital‐based cohort of Japanese patients with type 2 diabetes in comparison with CVD or end‐stage renal disease (ERSD).

## Methods

### Study participants

The Fukuoka Diabetes Registry includes 5,131 outpatients who were regularly followed in 16 diabetes specialist clinics in Fukuoka Prefecture, Japan (UMIN Clinical Trial Registry 000002627)^16^. The participants were registered between April 2008 and October 2010. Exclusion criteria were: (i) patients aged <20 years; (ii) those with drug‐induced diabetes; (iii) those with ESRD under dialysis; and (iv) those with serious diseases other than diabetes mellitus, such as cancer. After excluding 208 patients with type 1 diabetes mellitus, the remaining 4,923 patients were enrolled in the current study. The study was approved by the Kyushu University institutional review board (approval number 290, date of approval 4 January 2008), and followed the ethics of the Helsinki declaration with written informed consent.

### Baseline evaluation

Diabetes duration, current smoking habits and current alcohol intake were checked at the baseline. Leisure‐time physical activity (LTPA) was calculated as metabolic equivalent hours per week using Ainsworth's methods^17^. Blood pressure in the sitting position, bodyweight and height were measured, and body mass index (BMI) was calculated. Information regarding medications including insulin was collected from the medical records. Hemoglobin A_1c_ (HbA_1c_) was determined by high‐performance liquid chromatography (Tosoh Corp., Tokyo, Japan), serum low‐density lipoprotein cholesterol, high‐density lipoprotein cholesterol and creatinine concentrations by enzymatic methods, and serum albumin by the bromocresol purple method. The estimated glomerular filtration rate was calculated based on serum creatinine using the equation proposed by the Japanese Society of Nephrology^18^. Chronic kidney disease was defined as an estimated glomerular filtration rate of <60 mL/min/1.73 m^2^. The Geriatric Nutritional Risk Index was calculated by using the following equation: Geriatric Nutritional Risk Index = (1.489 × albumin [g/dL]) + (41.7 × [bodyweight / ideal bodyweight])^19^. The bodyweight / ideal bodyweight ratio was set to 1 when the patient's bodyweight exceeded the ideal bodyweight calculated from a BMI of 22 kg/m^2^ as its definition^20^.

### Mortality follow up

The primary outcome of the present study was all‐cause death. All participants underwent an annual follow up by interview, medical record review, telephone, letters and municipal registration of residence. A total of 27 participants were lost to follow up during the follow‐up period (median 5.3 years; follow‐up rate 99.5%). The underlying cause of death was determined based on the medical records and/or death certificate, and coded according to the International Classification of Diseases, 10th revision.

### Assessment of fractures, CVD and ESRD

Information regarding the history of fractures and CVD was obtained using a self‐administered questionnaire at enrollment, and the occurrence of fractures, CVD and ESRD was checked annually using a self‐administered questionnaire or by reviewing the medical records during the follow‐up period. CVD and ESRD were confirmed by contacting the participants’ attending specialists. Participants with hip fracture, upper limb fracture or CVD were defined as those with a history of the events at baseline or newly diagnosed events during the follow‐up period. CVD was defined as coronary heart disease and stroke. Participants with ESRD were defined as those who had started renal replacement therapy or died of ESRD during the follow‐up period.

### Statistical analysis

Differences in the mean values or proportions at baseline were assessed by Student's *t*‐test or the χ^2^‐test, as appropriate. Mortality was calculated in participants with or without hip fracture, upper limb fracture, CVD or ESRD using the person‐years method, and adjusted for age and sex by the direct method using 10‐year age groups. We evaluated the associations between the presence of hip fracture, upper limb fracture, CVD, ESRD and all‐cause death by logistic regression analysis and estimated odds ratios (ORs), and 95% confidence intervals (CIs). The multivariate‐adjusted model included age, sex, diabetes duration, BMI, current smoking habits, current drinking habits, LTPA, HbA_1c_, systolic blood pressure, low‐density lipoprotein cholesterol and insulin therapy. In our evaluation of the association between the presence of hip fracture and all‐cause death, we further adjusted for the presence of CVD and ESRD. Differences in the proportions of causes of death were evaluated by Fisher's exact test. All statistical analyses were carried out with Statistical Analysis Software (SAS) version 9.4 (SAS institute Inc., Cary, NC, USA). Values of *P *<* *0.05 were considered statistically significant in all analyses.

## Results

### Baseline characteristics

The baseline characteristics of participants with and without the presence of hip fracture, upper limb fracture, CVD and ESRD are shown in Table 1. In total, 110 participants had hip fractures, 801 had upper limb fractures, 1,344 had CVD and 104 had ESRD. Patients with hip fractures or CVD were older than those without hip fractures or CVD, but patients with upper limb fractures were younger than those without hip fractures or CVD. The proportion of male patients was lower among those with hip fractures than without hip fractures, but higher among those with CVD or ESRD than those without CVD or ESRD. The BMI was lower in patients with hip fractures than those without hip fractures, and higher in patients with upper limb fractures than those without upper limb fractures. The prevalence of current drinkers was lower among patients with hip fractures or ESRD than those without hip fractures or ESRD. LTPA was lower in patients with ESRD than those without ESRD. The HbA_1c_ level was higher in patients with upper limb fractures or CVD than those without upper limb fractures or CVD. The low‐density lipoprotein cholesterol level was lower in patients with hip fracture or CVD than those without hip fracture or CVD. The high‐density lipoprotein cholesterol level was lower in patients with than without CVD or ESRD. The prevalence of chronic kidney disease was higher in patients with hip fracture, CVD or ESRD than those without hip fracture, CVD or ESRD. Systolic blood pressure was higher in patients with CVD or ESRD than those without CVD or ESRD. Diastolic blood pressure was lower in patients with CVD than those without CVD. The prevalence of insulin users was higher among patients with hip fracture, CVD or ESRD than those without hip fracture, CVD or ESRD.

**Table 1:** Baseline characteristics of participants according to the presence of hip fracture, upper limb fracture, cardiovascular disease and end‐stage renal disease

	Hip fracture		Upper limb fracture		CVD		ESRD	
	(−)	(+)	(−)	(+)	(−)	(+)	(−)	(+)
*n*	4,813	110	4,122	801	3,679	1,344	4,819	104
Age (years)	65 ± 10	71 ± 7***	66 ± 10	64 ± 12***	64 ± 11	69 ± 8***	65 ± 10	65 ± 11
Male (%)	57	45**	57	57	53	66***	56	74***
Duration of diabetes (years)	16 ± 11	19 ± 11***	16 ± 11	15 ± 10	15 ± 10	18 ± 11***	16 ± 11	19 ± 10**
BMI (kg/m^2^)	23.8 ± 3.8	22.2 ± 3.3***	23.7 ± 3.8	24.0 ± 3.8*	23.7 ± 3.9	23.9 ± 3.5	23.8 ± 3.8	23.9 ± 3.6
Current smoker (%)	19	14	18	21	19	18	19	21
Current drinker (%)	40	20***	39	40	39	39	39	25**
LTPA (METs·h/week)	12 ± 15	11 ± 14	12 ± 15	12 ± 16	12 ± 15	11 ± 14	12 ± 15	9 ± 11**
HbA_1c_ (%)	7.43 ± 1.04	7.49 ± 1.10	7.42 ± 1.04	7.53 ± 1.09**	7.40 ± 1.02	7.53 ± 1.09***	7.44 ± 1.04	7.28 ± 1.23
HbA_1c_ (mmol/mol)	58 ± 11	58 ± 12	58 ± 11	59 ± 12**	57 ± 11	59 ± 12***	58 ± 11	56 ± 13
LDL cholesterol (mmol/L)	2.9 ± 0.7	2.7 ± 0.7*	2.9 ± 0.7	2.8 ± 0.7	2.9 ± 0.7	2.7 ± 0.7***	2.9 ± 0.7	2.7 ± 0.8
HDL cholesterol (mmol/L)	1.5 ± 0.4	1.5 ± 0.4	1.5 ± 0.4	1.5 ± 0.4	1.5 ± 0.4	1.4 ± 0.4***	1.5 ± 0.4	1.3 ± 0.4***
Serum albumin (g/dL)	4.4 ± 0.4	4.3 ± 0.4***	4.4 ± 0.4	4.4 ± 0.3	4.4 ± 0.3	4.3 ± 0.4***	4.4 ± 0.3	3.7 ± 0.5***
GNRI	106 ± 6	103 ± 7***	106 ± 6	106 ± 6	106 ± 6	105 ± 6***	106 ± 6	96 ± 8***
eGFR <60 mL/min/1.73 m^2^	21	31*	22	19	17	33***	20	89***
SBP (mmHg)	131 ± 17	128 ± 18	131 ± 17	131 ± 17	130 ± 17	133 ± 17***	130 ± 17	140 ± 25***
DBP (mmHg)	75 ± 11	71 ± 12**	74 ± 11	75 ± 11	75 ± 11	74 ± 11*	74 ± 11	74 ± 13
Insulin therapy (%)	29	42**	29	30	26	36***	28	77***

Values are expressed as the mean ± standard deviation or percentage. *P <* *0.05, **P <* *0.01, ***P <* *0.001. BMI, body mass index; CVD, cardiovascular disease; DBP, diastolic blood pressure; eGFR, estimated glomerular filtration rate; ESRD, end‐stage renal disease; GNRI, Geriatric Nutritional Risk Index; HbA_1c_, hemoglobin A_1c_; HDL, high‐density lipoprotein; LDL, low‐density lipoprotein; LTPA, leisure‐time physical activity; METs, metabolic equivalents; SBP, systolic blood pressure.

### Mortality with hip fracture, upper limb fracture, CVD and ESRD

**Figure 1:**
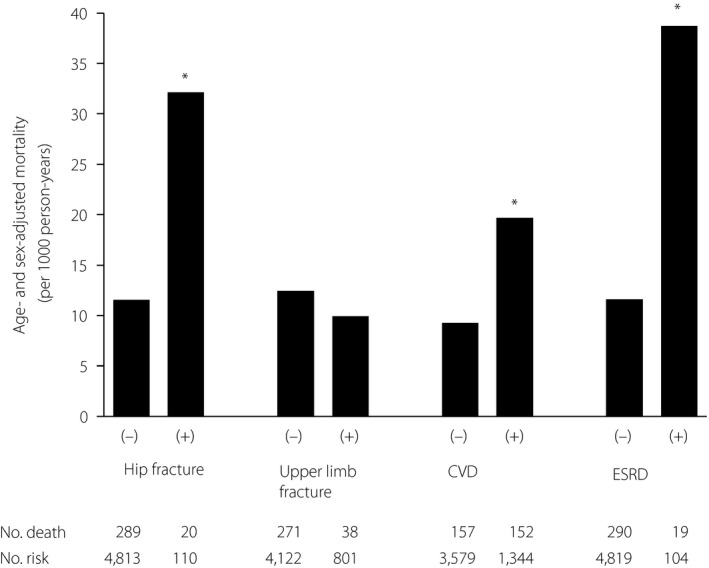
Age‐ and sex‐adjusted mortality in participants with or without hip fracture, upper limb fracture, cardiovascular disease (CVD), or end‐stage renal disease (ESRD). *P < 0.001 compared with those without.

Figure 1 shows the age‐ and sex‐adjusted mortality in participants with or without hip fracture, upper limb fracture, CVD or ESRD. The ORs for all‐cause mortality of hip fracture, upper limb fracture, CVD or ESRD are shown in Table 2. The age‐ and sex‐adjusted ORs were significantly higher in those with than without hip fracture, CVD and ESRD, but not in those with upper limb fracture. The multivariate‐adjusted ORs were significantly higher in patients with hip fracture, CVD and ESRD than those without hip fracture, CVD and ESRD (hip fracture OR 2.67, 95% CI 1.54–4.41; CVD OR 1.78, 95% CI 1.39–2.27; ESRD OR 2.36, 95% CI 1.32–4.05). In addition, further adjustment for the presence of CVD and ESRD did not alter the significance in patients with hip fracture (OR 2.74, 95% CI 1.58–4.54).

**Table 2:** Odds ratios and 95% confidence intervals for all‐cause mortality in participants with hip fracture, upper limb fracture, cardiovascular disease and end‐stage renal disease

	Hip fracture		Upper limb fracture		CVD		ESRD	
	(−)	(+)	(−)	(+)	(−)	(+)	(−)	(+)
Unadjusted	1.00 (Ref.)	3.48 (2.06–5.61)	1.00 (Ref.)	0.71 (0.49–0.99)	1.00 (Ref.)	2.78 (2.20–3.51)	1.00 (Ref.)	3.49 (2.04–5.70)
Age‐ and sex‐adjusted	1.00 (Ref.)	2.99 (1.75–4.89)	1.00 (Ref.)	0.79 (0.55–1.11)	1.00 (Ref.)	2.03 (1.59–2.58)	1.00 (Ref.)	3.31 (1.89–5.52)
Multivariate‐adjusted	1.00 (Ref.)	2.67 (1.54–4.41)	1.00 (Ref.)	0.77 (0.53–1.10)	1.00 (Ref.)	1.78 (1.39–2.27)	1.00 (Ref.)	2.36 (1.32–4.05)
Multivariate‐adjusted + CVD + ESRD	1.00 (Ref.)	2.74 (1.58–4.54)						

Multivariate‐adjusted model: age, sex, duration of diabetes, body mass index, current smoking habits, current drinking habits, leisure‐time physical activity, hemoglobin A_1c_, systolic blood pressure, low‐density lipoprotein cholesterol, and insulin therapy. CVD, cardiovascular disease; ESRD, end‐stage renal disease.

### Cause of death

**Figure 2:**
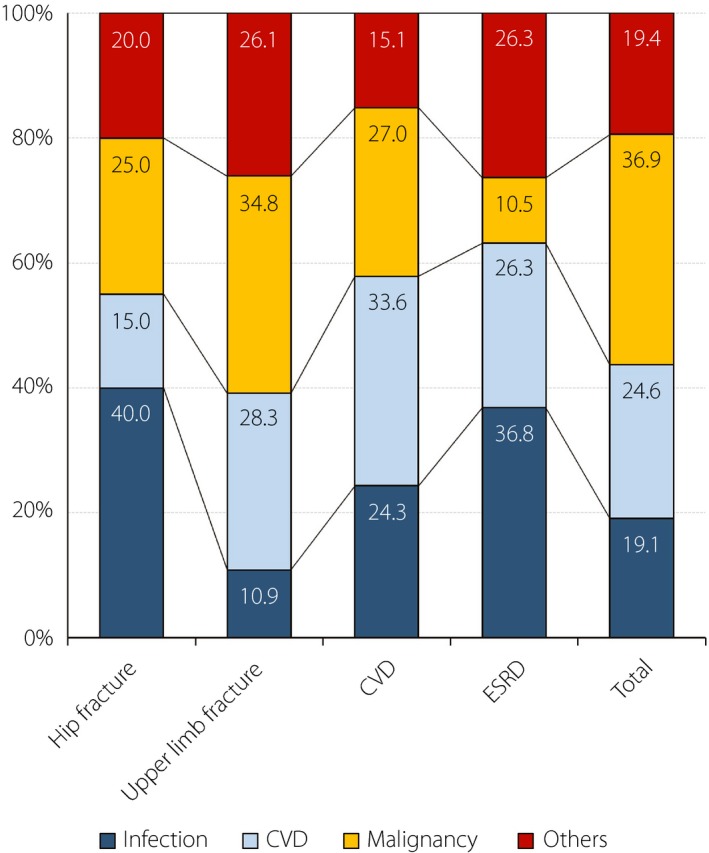
Main causes of death after hip fracture, upper limb fracture, cardiovascular disease (CVD) and end‐stage renal disease (ESRD), and that in all participants. Dark blue, infection; light blue, CVD; yellow, cancer; red, other diseases.

As shown in Figure 2, the main cause of death was infection among participants with hip fracture (40%) and ESRD (37%), cancer among those with upper limb fracture (35%) and in total (37%), and CVD among those with CVD (34%). The difference in the distribution of cause of death was insignificant (*P *=* *0.09). However, the proportion of patients with infection‐related death was significantly higher among those with hip fracture than those without hip fracture (*P *=* *0.03).

## Discussion

In the present study, the presence of hip fracture was associated with an increased risk of death among Japanese patients with type 2 diabetes. This association was significant, even after adjustment for potential confounders including BMI, smoking habit, duration of diabetes, HbA_1c_, physical activity, CVD and ESRD. In contrast, there was no significant association between upper limb fracture and all‐cause death. Furthermore, the magnitude of the impact of hip fracture on mortality appears to have been greater than that of CVD or ESRD, which are significant risk factors for death in patients with type 2 diabetes.

The risk of mortality persistently increases after hip fracture^21^. In previous research, the magnitude of the influence of hip fracture on all‐cause mortality was reflected by a hazard ratio (HR) of 2.12 among 122,808 participants from eight cohorts in Europe and the USA, with 4,273 incident hip fractures and 27,999 deaths during a mean of 12.6 years. Higher mortality might be caused by older age, dementia, frailty and postoperative complications, such as CVD and infection, which are common in patients with type 2 diabetes. The increased post‐fracture mortality in patients with type 2 diabetes was first reported in a study of the Spanish population in 2017 (type 2 diabetes, *n* = 3,861; non‐diabetic, *n* = 6,616)^22^. The effect of hip fracture on mortality was significantly higher in patients with type 2 diabetes (HR 2.90) than in those without (HR 2.59) independent of age, sex, BMI, smoking, alcohol intake and history of CVD, thus indicating increased mortality in association with type 2 diabetes (HR 1.28). The magnitude of the relative risk for death was similar to the present results (multivariable‐adjusted OR 2.67).

Four possible mechanisms for the increased mortality in patients with type 2 diabetes with hip fracture in the present study are as follows. First, patients with hip fractures were 6 years older than those without. However, the adjustment for age did not affect the mortality. Second, patients with hip fractures might be physically inactive. However, the registered participants in the present study were attending an outpatient clinic; they were not nursing home residents. Additionally, LTPA was not different between those with and without hip fractures. Third, there were no significant differences in risk factors for fracture, such as glycemic control, blood pressure and smoking. Furthermore, the adjustment for chronic kidney disease and insulin use did not affect the mortality. Finally, malnutrition might contribute to increased mortality in patients with hip fractures. The BMI, serum albumin level and Geriatric Nutritional Risk Index were significantly lower in patients with hip fractures than those without hip fractures. Malnutrition might lead to sarcopenia and induce falls and hip fractures, which could become complicated by fatal infection. In the present study (Figure 2), the leading cause of death was infection among patients with hip fracture (40%), although the leading cause of death was cancer in the total cohort (37%), which was in agreement with the Japanese Report of the Committee on Causes of Death in Diabetes Mellitus (38%)^23^. A recent study in Taiwan showed that the incidence of infectious disease was significantly higher in post‐fracture patients with type 2 diabetes than those without type 2 diabetes (OR 1.48 for urinary tract infection, OR 1.42 for septicemia and OR 1.13 for pneumonia)^24^.

The presence of CVD is significantly associated with an increased risk of death. All‐cause mortality of CVD was compared with that of hip fracture in 1,109 hospitalized patients in a community in Italy^25^. The age‐adjusted mortality rate was 14.5/100 person‐years after hip fracture, 14.3/100 person‐years after stroke, 6.9/100 person‐years after myocardial infarction without coronary revascularization and 2.0/100 person‐years after myocardial infarction with coronary revascularization. Fatality after stroke or myocardial infarction has been declining because of advancements in therapy in recent years^26^, ^27^. These findings were compatible with those in the present study; the OR for death was 2.67 in patients with hip fracture and 1.78 in those with CVD.

Patients with ESRD are usually treated with dialysis in Japan, and the prognosis has been poor according to the Japanese Society for Dialysis Therapy (3‐year survival rate of 65% in those who started dialysis in 1992, and 73% in those who started dialysis in 2006)^28^. Coco and Rush^29^ reported that the 1‐year mortality rate of hemodialysis patients with hip fractures was 2.7‐fold higher than that of hemodialysis patients without hip fractures, and 2.4‐fold higher than that in the general population with hip fractures. In the present study, however, the impact of hip fracture on mortality was not attenuated after adjusting for the presence of ESRD.

Previous studies have shown that mortality after forearm fractures is the same^30^, ^31^ or less^32^ than that in the general population. In a Swedish prospective cohort study of 2,847 patients with a low‐energy fracture at enrollment, the proportion of surviving patients was lower for hip fractures (41%) than for forearm (74%) or shoulder (64%) fractures at 5 years^9^. The European Prospective Osteoporosis Study showed that limb fracture was not correlated with aging, unlike hip fracture^33^. In the present study, there was no significant association between upper limb fracture and all‐cause death.

The main strength of the present study is the high follow‐up rate of death (99.5%), which enabled us to accurately determine the associations of hip fracture or other diabetes‐related complications with death against the background that the follow up of patients with severe comorbidities might be difficult. Furthermore, the cohort in the present study included potential confounders, such as physical activity, smoking habit, laboratory data and medications, and has been used to study fracture risks in patients with type 2 diabetes^34^, ^35^, ^36^.The present study also has several limitations. First, we derived incident fractures from self‐reported data, which might have resulted in misclassification. However, when the accuracy of the self‐administered questionnaire was evaluated in 455 fracture events by comparison with medical records, the agreement rate was 93.0%^34^. Furthermore, in the Women's Health Initiative Clinical Trial and Observational Study cohort, the validity of self‐reports for hip fracture was higher than that for other sites of fracture^37^. Second, because all participants in the current study were Japanese, whether the conclusions of the present study can be generalized to other ethnic populations remains unclear. In particular, the incidence of CVD is lower in Japanese than Western populations^38^. Third, because the current study was observational in nature, other confounding factors, besides those used in the study, might have been present.

The present study showed that the presence of hip fracture was associated with an increased risk of death among Japanese patients with type 2 diabetes, independently of CVD and ESRD. It should be emphasized that hip fracture is a critical event in the aging population of patients with type 2 diabetes during the present era of a better prognosis of CVD. In addition, whether prevention of hip fracture might improve the survival of patients with type 2 diabetes remains to be determined.

## Disclosure

The authors declare no conflict of interest.
